# The Meeting in Savannah

**Published:** 1895-05

**Authors:** 


### *EDITORIAL.*

The office of The Journal is in room 330 Equitable Building.

The editors are not responsible for views expressed by contributors.

Articles for publication should reach this office not later than the 15th instant.

Address all communications, and make all remittances payable to The Atlanta Medical and Surgical Journal, Atlanta, Ga.

Reprints of articles will be furnished, when desired, at a nominal cost. Requests for the same should always be made *on the manuscript.*

### THE MEETING IN SAVANNAH.

The Medical Association of Georgia met in Savannah on April 17, 18, and 19. The principal features of this meeting were the discussion of the State Lunatic Asylum, situated at Milledgeville, the expulsion of Dr. W. L. Bullard, of Columbus, and the substitution of a boat ride for a banquet. See report of the transactions in this issue.

As the paper of Dr. F. M. Ridley, the president-elect of the Association, will appear in the transactions of the Association with the title of "A Duty of the State Medical Association," it may be better to refer the reader to this source for his information in regard to the recommendations as to the Asylum therein contained. It is unfortunate that the discussion assumed the shape which it did, and especially so in view of the fact that Dr. T. O. Powell, Superintendent of the Georgia Lunatic Asylum, was called off to see a patient's family on the morning when Dr. Ridley read his paper.

The writer has had occasion to note the working of the Alabama Insane Hospital under the able management of the late Dr. Peter Bryce, and was impressed with the advantage to be derived from the absence of politics or of the meddlesome maneuverings of boards of trustees or of visitors.

Dr. Bryce took charge of the Alabama Insane Hospital at the age of twenty-six, and made the Hospital his life work of thirty-two years. The trustees, seeing that the right man was in the right place, became his co-operators, and yet left all the suggestions in the management of the hospital to him as the medical superintendent, their part being to indorse his suggestions. The Alabama Bryce Insane Hospital is probably the best conducted institution of its kind in the South, and ranks among the best in the world. The State of Georgia may well profit by the experience of our sister State and leave the supervision of the asylum in the hands of Dr. T. O. Powell, who has by experience and natural ability shown himself worthy of the high trust imposed upon him as superintendent.

The final responsibility must rest upon the superintendent, for there can be no person so well qualified to supervise the insane, and there can be no board of trustees or visitors so much interested in the welfare of the institution, as the staff of physicians whose existence is passed within its walls.

The Committee on Asylum Legislation reported last year that the legislature had appropriated $100,000 for an addition to the building. As the new building will probably be complete by the end of this year, let us hope that the Georgia Lunatic Asylum will become the equal of any institution of the kind in the world.

It is especially to be hoped that the efforts of the Association, begun nearly ten years ago, and renewed at this meeting, may be successful in the establishment of an inebriate asylum.

The desire of the Medical Association to sustain the Code of Ethics which has been its guide for so many years is evidenced by the unanimous adoption of the report of the board of censors in regard to the use of cuts in the newspapers, and of reading notices and bids for practice, and it is to be hoped that the standard of the medical profession in Georgia may be upheld at all hazards.

The entertainment of the members, both individually and collectively, could not have been more complete and satisfactory. Drs. M. L. Boyd and W. H. Elliott, of the committee of arrangements, are to be congratulated on the plan they so brilliantly carried out for the pleasure and profit of all present.

The DeSoto, which was headquarters for the Association, is one of the finest hotels in the South, and added greatly to the comfort of all. The street-car ride to Thunderbolt and Isle of Hope was also greatly appreciated. But the crowning glory of the whole plan was the excursion to Wilmington on the beautiful steamboat, "The Vigilant." The day was ideal, and the presence of ladies was no small item in the sprightliness and the rapture of a boat ride.

With handkerchiefs flying and with the banner to the breeze, we left the wharf at one o'clock returning at dark. The banks of oysters in their shells awaiting us at Wilmington were gradually made less as shovelful after shovelful passed from the fire to the numerous tables surrounded by eager faces.

The looker-on would imagine that the hearty and solemn discussions on appendicitis and kindred topics had been forgotten, and that the doctors were boys again. J. McF. G., Jr.

